# Multimodal Large Language Models for Dental Chart Image Interpretation: Cross-Sectional Benchmarking Study With Students and Clinicians

**DOI:** 10.2196/91809

**Published:** 2026-09-04

**Authors:** Ah-Young Cho, Soo-Heang Eo, Mi-Jeong Jeon, Jae-Hoon Kim, Jungjoon Ihm, Deog-Gyu Seo

**Affiliations:** 1 Department of Conservative Dentistry and Dental Research Institute, School of Dentistry, Seoul National University, 101 Daehakno, Jongno-Gu, Seoul, 03080, Republic of Korea, 82 2-6256-3182; 2 Graduate School, Department of Urban Big Data Convergence, University of Seoul, Seoul, Republic of Korea; 3 AI Agent Team, CryptoLab Inc., Seoul, Republic of Korea; 4 Department of Conservative Dentistry and Oral Science Research Center, Yonsei University College of Dentistry, Seoul, Republic of Korea; 5 Department of Dental Education, Dental and Life Science Institute, School of Dentistry, Pusan National University and Dental Research Institute, Busan, Republic of Korea; 6 Department of Dental Education and Dental Research Institute, School of Dentistry, Seoul National University, Seoul, Republic of Korea

**Keywords:** dental records, education, dental, students, dental, clinical competence, large language models, generative AI, ChatGPT, multimodal ChatGPT, benchmarking, natural language processing

## Abstract

**Background:**

Entering clinical training, dental students must learn to read tooth-centered electronic dental records, but limited teaching time in patient-centered clinics can leave gaps in chart-reading literacy. Multimodal large language models (LLMs) that process dental record images may offer scalable educational support, yet their performance has not been benchmarked.

**Objective:**

This study evaluated multimodal ChatGPT models on dental chart image interpretation and compared the best-performing model with dental students and residents.

**Methods:**

A retrospective, cross-sectional benchmark study used deidentified dental chart images from 15 patients at Seoul National University Dental Hospital (2017‐2025). Charts containing Korean and English text were captured as sequential screenshots (154 images). For each patient, 24 Korean-language questions (360 total) spanned four categories: Type A, general factual retrieval; Type B, tooth- or procedure-specific retrieval; Type C, interpretation requiring multientry synthesis; and Type D, absent-information questions assessing abstention. Nine multimodal ChatGPT models (available August 2, 2025‐August 9, 2025) were evaluated under standardized conditions. Outputs were scored against a gold standard using 7 metrics, with sentence bidirectional encoder representations from transformers (SBERT) similarity prespecified as the primary semantic measure. Human baselines included 2 third-year students and 2 first-year residents. Groups were compared with Kruskal-Wallis tests and Dunn post hoc analyses. Gold-standard reliability was assessed by independent senior-expert review and chance-corrected agreement (Gwet AC1) among clinical reference raters, and the model ranking was confirmed by content-based clinical accuracy analysis.

**Results:**

Across 360 items, GPT-5 Thinking achieved the highest median SBERT similarity (0.900, IQR 0.525‐1.000), followed by GPT-5 Pro (0.861, IQR 0.501‐1.000) and OpenAI o3 (0.831, IQR 0.489‐1.000), with significant overall group differences (*P*<.001). Student 1 did not differ significantly from GPT-5 Thinking across Types A-D (all *P*≥.16), whereas Student 2 differed only on Type D (*P*=.002; Cliff δ=−0.27). Resident 1 scored higher than GPT-5 Thinking on Types A (*P*=.01) and C (*P*=.04) but lower on Type D (*P*<.001; δ=−0.35), whereas Resident 2 scored higher on Types A (*P*=.03), B (*P*=.02), and C (*P*=.04) and did not differ on Type D (*P*=.23). Type C tasks showed compressed SBERT distributions and low exact-match rates, indicating persistent difficulty in synthesis. The clinical reference rater agreement was high (Gwet AC1=0.99), and the content-based clinical-accuracy ranking was closely aligned with the SBERT ranking (Spearman ρ=0.90; *P*=.001).

**Conclusions:**

Statistically nonsignificant differences were observed between GPT-5 Thinking and dental students for most question-type contrasts, with Student 2 differing only on absent-information items. Compared with first-year residents, GPT-5 Thinking remained lower on several Type A-C contrasts, particularly interpretive Type C and one tooth- or procedure-specific Type B comparison; Type D contrasts require cautious interpretation because all groups had ceiling medians. Within this single-center benchmark, multimodal LLMs may have potential as supervised educational tools for chart-reading practice and verification, rather than as replacements for clinical expertise, pending external validation across institutions, specialties, and record systems.

## Introduction

At the preclinical-to-clinical transition, when learners first enter the clinical environment, dental students must develop the ability to read and interpret dental charts, also referred to as dental records, including locating relevant information, reconstructing treatment timelines, and distinguishing tooth-specific details from patient-level records [1,2]. For students new to clinical practice, this process can be challenging and may add cognitive load in already demanding learning environments, particularly within patient-centered clinics where clinical requirements limit protected teaching time [3,4]. Although dental charts are foundational records of a patient’s history and guide subsequent treatment, limited clinical exposure can lead to chart misinterpretation, increasing the downstream risk of treatment errors [5,6]. Because this transition is formative, curricula should minimize gaps by providing explicit early instruction and structured guidance to support competence development. However, current dental curricula often prioritize hands-on procedural training, leaving the cognitive skills required for interpreting clinical records largely implicit [4]. The volume and complexity of chart data, coupled with the clinical priorities of patient-centered care, further constrain opportunities for timely teaching and feedback. Ideally, faculty would regularly assess students’ chart-reading skills and provide targeted coaching; in practice, patient-care responsibilities limit real-time instruction, and scalable assessment of comprehension is rarely feasible. Consequently, developing “chart-reading literacy”—the ability to locate, interpret, and synthesize information from complex dental charts—represents a core educational objective and a prerequisite for clinical reasoning and procedural competence in dentistry [1-4].

Against this backdrop, large language models (LLMs) have emerged as potential educational aids. LLMs can retrieve, organize, and synthesize information into coherent, task-focused explanations, and recent advances in training and alignment have substantially improved their accuracy and robustness [7,8]. ChatGPT, in particular, is a widely renowned LLM encompassing multiple releases, including reasoning-focused models and the GPT-4 series; most recently, the GPT-5 family (GPT-5, GPT-5 Thinking, and GPT-5 Pro) has expanded multimodal capabilities and strengthened reliability and reasoning. Learners across disciplines, including dental students, increasingly use ChatGPT to seek information and clarify unfamiliar topics [9,10]. Its chat-based interface supports AI-guided learning by enabling users to pose questions, test interpretations, and iteratively refine understanding through interactive dialogue. For students navigating the preclinical-to-clinical transition, ChatGPT may facilitate verification of chart interpretations, provide immediate clarification, and model structured reasoning in real time. Consequently, generative AI-guided education has been proposed as an emerging approach in dental education, positioning LLMs as adaptive instructional partners rather than passive information sources [11]. At the same time, persistent limitations—including hallucinations, sensitivity to prompt formulation, and variable performance across task types—highlight the continued need for supervision and verification [12,13].

Dental electronic records present distinct interpretive challenges that distinguish them from general electronic medical records (EMRs). Whereas most EMRs are organized around patient-level narrative entries, dental records are organized at the level of individual teeth and integrate spatial elements such as odontograms, tooth-numbering grids, and procedure maps [14]. Relevant information is frequently distributed across multiple pages and time points so that even straightforward tasks, such as reconstructing the treatment history of a single tooth, require learners to integrate spatial cues with textual entries [5,6,14]. Critically, these spatial cues cannot be faithfully preserved when dental records are linearized into text alone, and text-only approaches therefore fail to capture the tooth-level context central to accurate interpretation of dental records [4,15]. This challenge is compounded by substantial heterogeneity and documentation gaps in dental EMRs, which prior work in dental informatics has highlighted in calls for dental-specific templates and standardization [6,16]. These characteristics indicate that meaningful evaluation of generative AI for dental chart reading requires multimodal inputs in which models reason directly over images of dental charts.

Despite this potential, few rigorous assessments have examined how generative AI models perform on dental chart images, particularly in educational contexts. Prior research in dental informatics has largely focused on coding systems, data quality, and secondary data use, with limited investigations into whether LLMs can accurately retrieve tooth-level information, reason across dispersed chart entries, and appropriately abstain when information is absent [14,15,17]. This gap is consequential because well-characterized tools could reduce barriers to chart comprehension, support deliberate practice in clinical reasoning, and free instructional time for supervised skill acquisition, whereas poorly characterized tools risk amplifying errors and fostering false confidence. If ChatGPT’s performance in educational settings is systematically validated, it may provide students navigating the preclinical-to-clinical transition with a scalable means to practice chart reading and verify their interpretations, even in the absence of formalized chart-reading instruction [11,15].

This study evaluated multimodal ChatGPT models using dental chart images as inputs. The official models evaluated were OpenAI o3, GPT-4.5, GPT-4.1, GPT-4.1 mini, OpenAI o4-mini, GPT-4o, GPT-5, GPT-5 Thinking, and GPT-5 Pro. Models were benchmarked using semantic and exact-match evaluation metrics across 4 question categories, including general factual retrieval, tooth- or procedure-specific retrieval, interpretive reasoning, and absent-information (unanswerable) tasks. The highest-performing model was subsequently compared with dental students at the preclinical-to-clinical transition and with resident clinicians to assess relative performance in an educational context. This study aimed to benchmark the chart-image interpretation performance of multimodal ChatGPT models across these 4 task categories and to compare the best-performing model with dental students at the preclinical-to-clinical transition and with qualified clinicians in order to characterize where such models responsibly support chart-reading education.

## Methods

### Study Design and Overview

**Figure 1.**
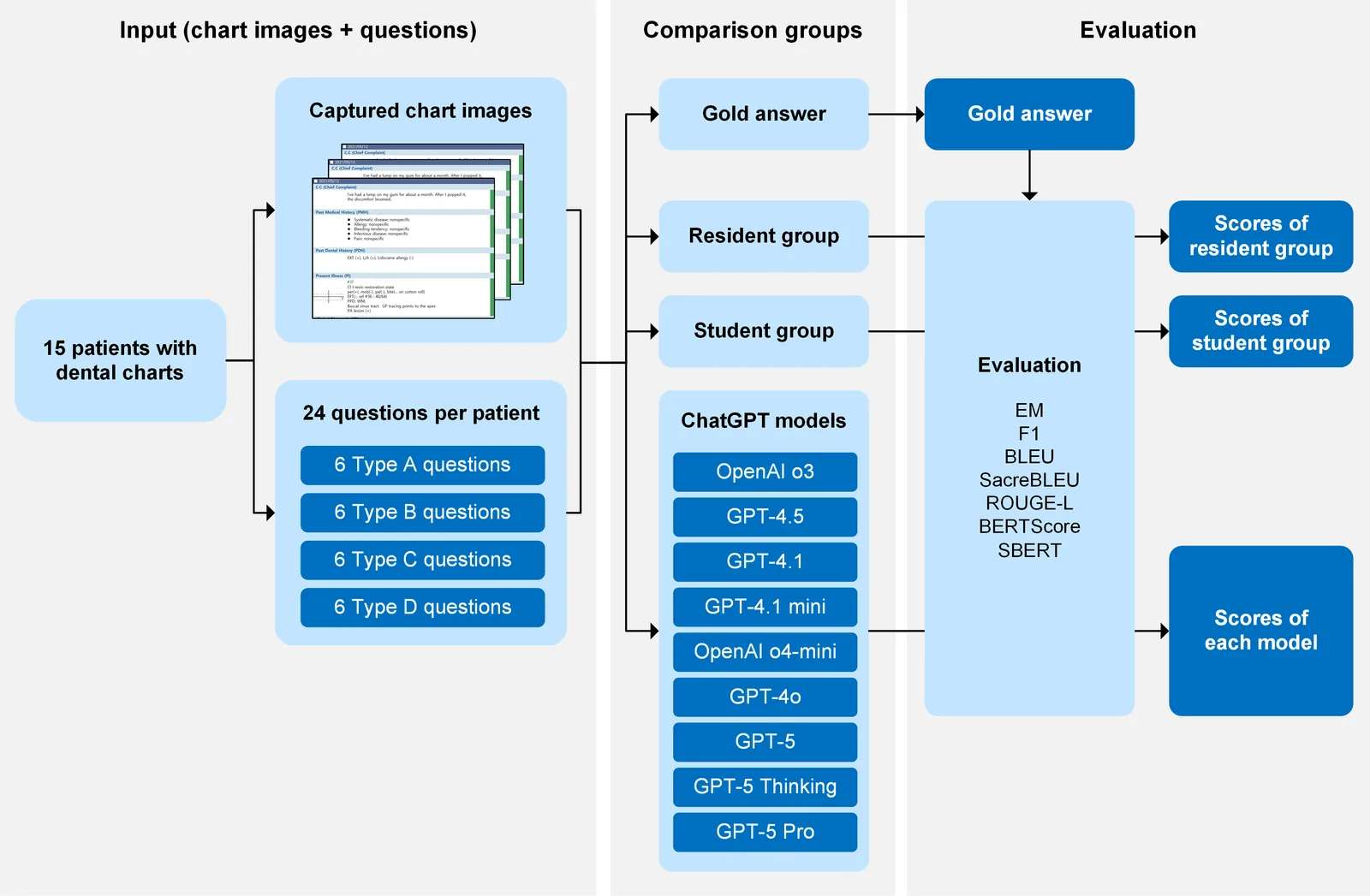
Flowchart of study design and evaluation process. BERT: bidirectional encoder representations from transformers; BLEU: bilingual evaluation understudy; EM: exact match; ROUGE-L: recall-oriented understudy for gisting evaluation-longest common subsequence; SBERT: sentence bidirectional encoder representations from transformers.

A retrospective, cross-sectional evaluation was conducted using deidentified dental chart images under standardized testing conditions. The reporting of this study followed the STROBE (Strengthening the Reporting of Observational Studies in Epidemiology) checklist (Checklist 1). The primary objective was to assess multimodal ChatGPT performance across three education-relevant competencies: (1) factual retrieval from dental charts, including general and tooth- or procedure-specific information, (2) inferential reasoning across dispersed chart entries, and (3) appropriate handling of missing or absent information. Analyses were prespecified and reported overall, by model, and by question type. The study workflow is summarized in Figure 1.

### Data Source and Case Selection

**Figure 2.**
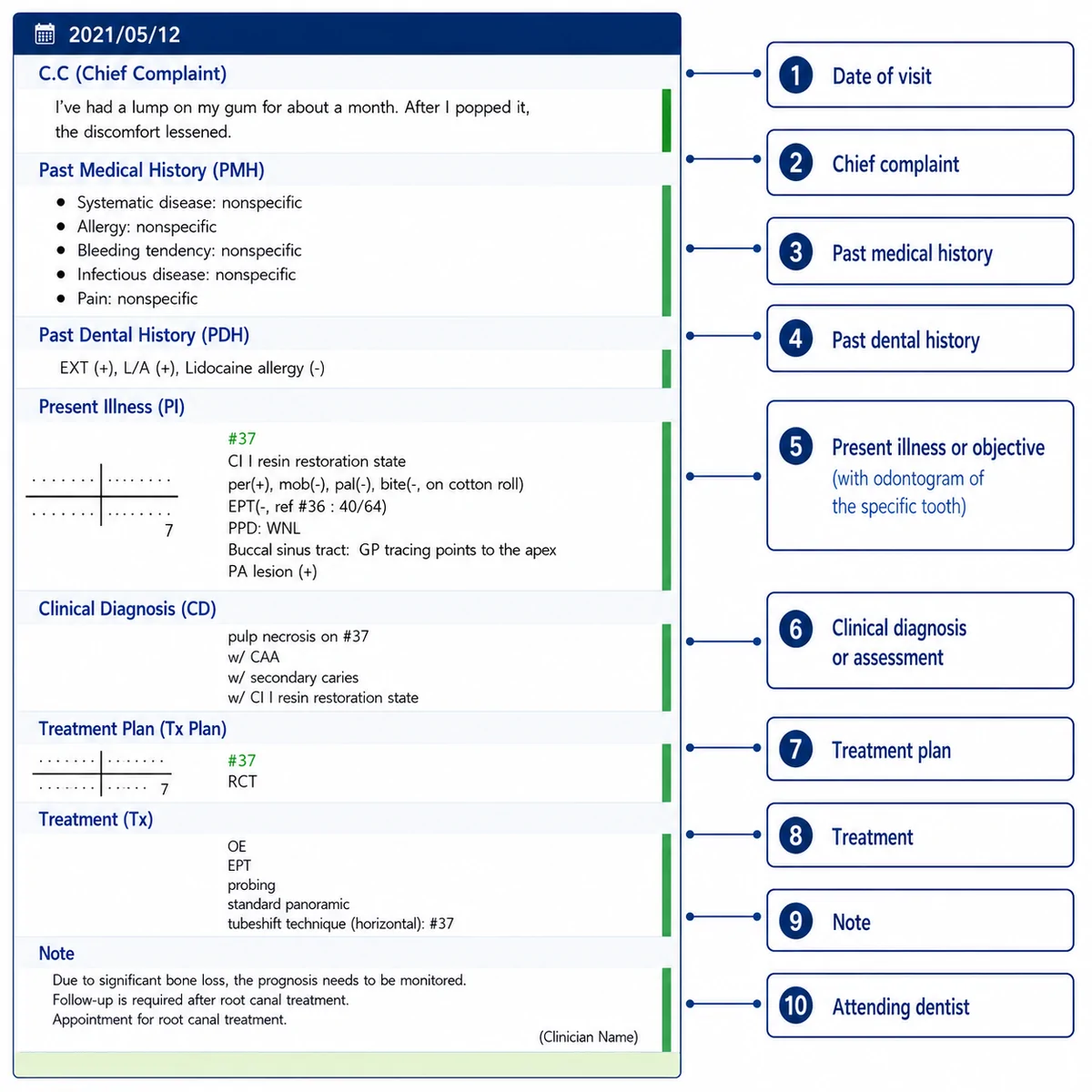
Example structure of an image-captured dental chart format. CAA: chronic apical abscess; EPT: electric pulp test; EXT: extraction; GP: gutta percha; L/A: local anesthesia; PA: periapical; PPD: probing pocket depth; WNL: within normal limits.

Dental charts from 15 patients were sampled from the Department of Conservative Dentistry at Seoul National University Dental Hospital (SNUDH), covering the period from January 1, 2017, to June 30, 2025. The charts contained both Korean and English text. Figure 2 presents an example of a captured dental chart image, including the patient’s chief complaint, past medical and dental histories, current dental problems (present illness) with associated odontograms, clinical diagnoses, treatment plans, treatments performed, and free-text clinical notes. The case mix comprised restorative (n=6), endodontic (n=5), surgical (n=2), and mixed or complex cases (n=2). Inclusion criteria were as follows: (1) documentation of treatment for at least one tooth, (2) the presence of chart elements verifiable from images (eg, odontograms, procedure notes, and visit dates), and (3) complete deidentification prior to data export. Charts containing any personally identifiable information were excluded. Where feasible, cases were selected from multiple providers to reduce operator-specific variability.

### Image Acquisition and Preprocessing

Dental charts were captured as sequential screenshots with a resolution of 717×742 pixels. Consecutive images overlapped by at least one line of text to prevent truncation at page boundaries. The number of images per patient ranged from 3 to 26 (mean 10.27, SD 6.54; total 154 images). Because the testing interface allowed a maximum of 10 images per message, larger cases exceeding this limit were uploaded in consecutive batches within the same conversation thread to preserve page order and context. All images were manually reviewed to ensure legibility and complete deidentification prior to use.

### Question Dataset and References

For each patient, 24 individualized questions in Korean were developed, resulting in a total of 360 questions. Questions were evenly distributed across 4 categories (Table 1):

- Type A, general (6 questions): explicit chart facts, such as dates and provider names. Three items were common across all cases: first-visit date, total number of visits, and primary provider name.
- Type B, dental (6 questions): tooth- or procedure-specific clinical details, for example, the date of a specific restoration or the presence of a final crown.
- Type C, interpretation (6 questions): inference questions requiring synthesis of evidence across multiple chart entries, such as the rationale for a procedure.
- Type D, absent information (6 questions): intentionally unanswerable questions designed to test appropriate abstention.

**Table 1.** Question types for dental chart evaluation: examples and rationale for large language model (LLM) assessment.

Category	Number of questions	Examples	Rationale for LLM^a^ evaluation
General	6	When is the first visit to the clinic?	Evaluation of basic recognition of simple information. Suitable for assessing OCR^b^ accuracy and fundamental contextual understanding.
Dental	6	When was tooth number 16 treated for a cavity?	Recognition of technical dental terms. Useful for testing the ability to extract relevant details and apply subject-specific understanding.
Interpretation	6	For what purpose was the MTA^c^ used for the treatment?	Assesses the ability to synthesize information from multiple sources and make informed inferences. Enables evaluation of integrated reasoning skills.
Absent information	6	What was the length of MB2^d^ canal in tooth number 11? (despite the absence of MB2 canal)	Checks whether the system avoids generating incorrect information not present in the clinical chart. Essential for evaluating hallucination control capabilities.

a LLM: large language model.

b OCR: optical character recognition.

c MTA: mineral trioxide aggregate.

d MB2: mesiobuccal 2.

The 4 question categories integrate Bloom taxonomy and established natural language processing (NLP) evaluation paradigms [18]. Types A and B assess foundational information retrieval via single-hop queries, distinguishing baseline reading comprehension (Type A) from dental-specific domain expertise (Type B). Type C evaluates higher-order clinical reasoning, requiring multihop inference across dispersed chart entries. Type D adapts the unanswerable questions paradigm from robust NLP benchmarks (eg, SQuAD 2.0 and emrQA [19]) to assess hallucination control and appropriate abstention, a critical safety prerequisite.

Questions were manually formulated by a single clinical researcher (AYC) to accurately reflect the unique clinical context of each patient case. Methodological consistency was ensured by anchoring all generated questions to the predefined conceptual framework (Types A, B, C, and D). As the primary objective of this study was to compare relative model performance, all evaluated models were presented with the identical set of case-specific questions, ensuring a fair and consistent comparative benchmark. The questions were authored by a single clinical researcher (AYC) and were not independently reviewed for wording prior to evaluation; however, the associated gold-standard answers were subsequently verified by an independent senior specialist.

Gold-standard answers were provided by a licensed dentist and full professor in the Department of Conservative Dentistry at SNUDH, with over 20 years of clinical experience. Two first-year residents from the same department contributed additional comparison answers. In this study, these residents were operationally defined as the “resident clinician group.” This terminology is justified as these individuals hold valid dental licenses and possess approximately 3-4 years of total clinical experience when accounting for their undergraduate clinical training. Consequently, they are considered capable of independent patient management and provided a formally qualified clinical baseline. For the student baseline, 2 third-year dental students at the preclinical-to-clinical transition (2 years of preclinical followed by 2 years of clinical curriculum) produced independent answer sets. This level was chosen to reflect students entering initial clinical exposure with limited prior experience in chart interpretation. All human raters completed the same set of 24 individualized questions per patient independently and without access to model outputs.

### Model Conditions and Inference Procedure

Nine ChatGPT models available from August 2-9, 2025, in Korea Standard Time via the ChatGPT (OpenAI) user interface were evaluated, namely OpenAI o3, GPT-4.5, GPT-4.1, GPT-4.1 mini, OpenAI o4-mini, GPT-4o, GPT-5, GPT-5 Thinking, and GPT-5 Pro. Evaluations were performed under a ChatGPT Pro subscription, the highest consumer tier available at the time. The study included all image-capable models selectable in the consumer interface during this window rather than a performance-based subset. Model inclusion was therefore governed by interface availability under this subscription tier; GPT-5 Pro, in particular, was accessible only at the Pro tier. For each patient-model pair, a new conversation was initiated to prevent cross-case contamination. All chart images for the patient, including continuation batches, and the corresponding 24 questions were presented together, accompanied by standardized Korean instructions directing the model to review all images and answer the questions. To ensure methodological transparency and reproducibility, the direct links to the complete conversational logs with the LLMs, including all exact prompts and generated responses, are provided in Multimedia Appendix 1. Conversation history was reset between models and between patients. Model temperature and other settings were left at platform defaults. All evaluated models support text-and-image inputs in ChatGPT; however, formal optical character recognition (OCR) accuracy metrics for these interfaces are not published. Context limits vary by model (approximately GPT-4.1 up to ~1 million tokens via API, GPT-5 up to ~400,000 tokens via API, and ~256,000 in ChatGPT). Comparative specifications of each model, including type, multimodal data support, context-window size, visual input handling, distinguishing features, and release date, are summarized in Table 2 [20-22]. The consumer interface was used to reflect how students and clinicians actually access these models, prioritizing ecological validity. The trade-off is that platform-level factors—proprietary system prompts, memory-state handling, rate-limiting behavior, and per-query routing decisions, including GPT-5 smart-routing—are not exposed and could not be independently verified or held under experimental control.

**Table 2.** Characteristics of ChatGPT models, including type, multimodal data support, context length, image-processing capability, key features, and release date.

AI model	Type	Data support	Context length	Image understanding performance	Key features	Release date
OpenAI o3	Reasoning	Text+image	Not disclosed	Supports visual input; no explicit OCR^a^ evaluation	Optimized for structured scientific and coding reasoning, cost-efficient reasoning engine	April 16, 2025
GPT-4.5	GPT	Text+image	128,000 tokens	Supports visual input; no explicit OCR evaluation	Enhanced natural, intuitive dialogue, and aesthetic creativity; reduced hallucination and emotional IQ boost	February 27, 2025
GPT-4.1	GPT	Text+image	1 million tokens	Supports visual input; no explicit OCR evaluation	Large context for vision tasks	April 14, 2025
GPT-4.1 mini	GPT	Text+image	1 million tokens	Same as above; no specific OCR info	Scaled-down version of GPT-4.1; cost-efficient for similar tasks	April 14, 2025
OpenAI o4-mini	Reasoning	Text+image	Not disclosed	Enhanced visual reasoning; OCR not specified	Compact model with speed and accuracy; excels in technical tasks and visual reasoning	April 16, 2025
GPT-4o	GPT	Text, image, audio, and video	128,000 tokens	Strong multimodal; OCR not formally measured	Multimodal (voice, vision, and translation) with state-of-the-art benchmarks	May 13, 2024
GPT-5 (default)	GPT	Text, image, audio, and video	256,000 tokens	Enhanced visual perception; no explicit OCR metrics	Smart routing (fast vs deep mode), high performance in coding, writing, health, and perception	August 7, 2025
GPT-5 Thinking	GPT	Text, image, audio, and video	256,000 tokens	Tuned for deep reasoning; no explicit OCR mention	Tuned for deeper, slower reasoning; selected via router or user prompt	August 7, 2025
GPT-5 Pro	GPT	Text, image, audio, and video	256,000 tokens	Highest compute capability; no explicit OCR performance claim	High-compute, extended reasoning variant best suited for complex tasks	August 7, 2025

a OCR: optical character recognition.

### Outcomes and Evaluation Metrics

For Type D items, which were designed to assess the ability to recognize missing information, the expert reference answer was standardized as “not recorded.” Because models expressed absence in heterogeneous ways (eg, “no record found” and “not noted in the chart”), simple lexical comparisons could underestimate correct abstention. To address this, all responses explicitly indicating a lack of information were manually normalized to “not recorded” prior to scoring. This normalization procedure was applied consistently across all models and human raters.

Model outputs were compared against the gold-standard answers. Seven complementary metrics, all normalized to a continuous scale ranging from 0 to 1, were computed to capture both exactness and semantic adequacy:

1. Exact match (EM): the percentage of predictions that exactly match the reference answers, representing a strict, all-or-nothing measure. Values are 0 or 1; for free-text answers, a median of 0 is expected, with 1 indicating verbatim matches.
2. Token-level *F_1_*-score: the harmonic mean of precision and recall calculated at the word (token) level, assessing overlap between predicted and reference text. Values range from 0 to 1, with values near 1 indicating close lexical overlap.
3. Bilingual evaluation understudy (BLEU): evaluates the quality of machine-generated text by measuring n-gram overlap between the prediction and a set of high-quality reference texts [23]. Values range from 0 to 1 (higher is better); values above ~0.30 are considered understandable, and above ~0.50 are good and fluent [24].
4. SacreBLEU: a standardized implementation of BLEU that ensures reproducible scores by handling tokenization and preprocessing consistently [25]. It shares BLEU’s 0‐1 scale and interpretation.
5. Recall-oriented understudy for gisting evaluation-longest common subsequence (ROUGE-L): measures the longest common subsequence between the generated and reference text, emphasizing recall [26]. Values range from 0 to 1; like other overlap metrics, it yields near-zero values for free-text answers.
6. Bidirectional encoder representations from transformers (BERT) score: computes semantic similarity between candidate and reference text by aligning words using contextual embeddings from BERT [27]. BERTScore values range from –1 to 1, although in practice values fall within the 0‐1 range; roughly, ≥0.90 is faithful, 0.66‐0.90 adequate, and ≤0.65 meaning-divergent.
7. Sentence-BERT (SBERT): a modification of BERT using Siamese and triplet network architectures to generate semantically meaningful sentence embeddings, suitable for large-scale semantic comparison tasks [28]. Cosine similarity ranges from −1 to 1 but effectively falls within 0-1; commonly cited semantic-similarity thresholds fall in the 0.5‐0.7 range.

### Statistical Analysis

All automatic evaluation metrics were computed against the gold-standard answers on a per-question basis, with all items weighted equally (macro-averaging).

For each ChatGPT model, item-level scores were calculated across all 360 questions from the 15 charts. Distributions of EM, token-level *F*_*1*_-score, BLEU, SacreBLEU, ROUGE-L, BERTScore, and SBERT were summarized per model using the median and IQR and visualized with boxplots. Model rankings and between-model comparisons were described descriptively.

To compare performance by task category, SBERT was prespecified as the primary metric. For each model and question type—A (general), B (dental), C (interpretation), and D (absent information)—item-level SBERT values were summarized using medians and IQRs and displayed as boxplots to facilitate within-type comparisons between models. This analysis included all 360 questions, with each item weighted equally.

For SBERT human-model comparisons, groups included Student 1, Student 2, Resident 1, Resident 2 (first-year residents), and the highest-performing GPT model. Analyses were conducted separately for question Types A-D. Because score distributions were nonnormal according to Shapiro-Wilk tests, overall group differences were assessed using the Kruskal-Wallis test, followed by Dunn post hoc tests with Holm-Bonferroni adjustment for pairwise contrasts of primary interest (each human group vs GPT-5 Thinking). All tests were 2-sided with a significance threshold of *α*=.05. Items with missing or empty reference answers were excluded listwise from the relevant comparisons. To convey practical significance alongside *P* values, effect sizes appropriate to the nonparametric design were computed: epsilon-squared (ε²) for each Kruskal-Wallis omnibus test and Cliff delta (δ) with 95% bootstrap CIs (10,000 resamples) for each prespecified pairwise contrast (each human group vs GPT-5 Thinking), interpreted with Cohen-equivalent benchmarks (ε²=0.01/0.06/0.14; |δ|=0.147/0.33/0.474 for the small/medium/large boundaries). SBERT embeddings for these comparisons were generated using sentence-transformers/paraphrase-multilingual-MiniLM-L12-v2.

Interrater reliability of the gold-standard answers was assessed in 2 ways. First, an independent second senior specialist (JHK) in Conservative Dentistry reviewed all 360 gold-standard answers against the source charts and rated each as clinically acceptable. Second, because the senior faculty member and the 2 residents had answered the same items independently, each free-text answer was reduced to its canonical clinical value, and chance-corrected agreement was computed—percent agreement, Krippendorff α (nominal), Fleiss κ, and Gwet AC1. Gwet AC1 was prespecified as the primary coefficient because κ can be unstable under near-ceiling agreement. Agreement was assessed overall and by question type for the clinical reference raters (gold standard and 2 residents) and, for transparency, for all 5 human raters. The canonical-value extraction was performed by an LLM used solely as an extractor (not as a judge of clinical correctness) and was validated against an independent dentist on a random 20% subset (50 items).

To assess whether the SBERT-based model ranking was not an artifact of embedding similarity, all 9 models were additionally scored for clinical correctness against the gold value (clinical content for Types A-C and appropriate abstention for Type D), and the resulting content-based clinical-accuracy ranking was compared with the SBERT ranking using Spearman rank correlation. As a safety analysis, the proportion of Type D items on which each model supplied a fabricated or unsupported value instead of abstaining was quantified. As a focused exploratory subanalysis, rather than a full multilingual evaluation, accuracy was compared between items whose expected answer was in Korean script and those whose expected answer was English or numeric, within a single task type. Because the charts contained both Korean and English within the same record, this comparison assessed the script of the expected answer rather than model performance across separate languages. All source code for model execution is available from the project’s GitHub repository [29].

To determine whether discrepancies flagged or missed by the automated metrics were clinically consequential, a prespecified expert-adjudicated subset analysis was performed for the 2 best-performing models. The adjudicated subset comprised all Type A-C responses scored as incorrect in the content-based clinical-accuracy rescoring (n=23), all Type D responses in which a model supplied a value instead of abstaining (n=6), and a random sample of 15 responses per model previously classified as content-correct with low SBERT similarity (<0.60; n=30), the last stratum serving to verify that low semantic similarity did not conceal clinical errors (59 responses in total). Two faculty dentists at a university dental hospital, neither of whom had been involved in question authoring or gold-standard construction, independently rated each response in randomized order, blinded to model identity, automated scores, and subset stratum, on a 3-level scale: 0, no clinical consequence; 1, minor discrepancy unlikely to alter interpretation or management in a supervised educational setting; and 2, clinically consequential, defined as plausibly changing chart interpretation or a management decision or teaching an incorrect clinical fact. The interadjudicator agreement was quantified using Gwet AC1 and linearly weighted Cohen κ, and disagreements were resolved by consensus.

All metric computations and statistical analyses were performed using Python (version 3.13.2; Python Software Foundation) on Windows 10 (Microsoft Corp).

### Exploratory Qualitative Feedback

As a secondary exploratory component, written qualitative feedback was collected from the 5 human participants (2 third-year dental students, 2 first-year residents, and the senior faculty dentist who established the gold standard) after they completed the chart-reading tasks. Participants responded in Korean to three open-ended questions concerning (1) the perceived advantages of AI-assisted dental chart interpretation for learning, (2) the perceived disadvantages or concerns, and (3) how the tool compared with traditional faculty-guided chart-reading instruction. Free-text responses were analyzed using a conventional content analysis approach. Two reviewers (MJJ and JI) independently read all responses, inductively coded recurrent ideas, and grouped them into themes for each question. The frequency of each theme was reported as the number of participants expressing it. Representative excerpts were translated from the original Korean and lightly edited for clarity. Because this component was exploratory and based on a small convenience sample, it was intended to contextualize, rather than to formally test, the quantitative benchmark findings. The full thematic summary and excerpts are provided in Table S1 in Multimedia Appendix 2.

### Ethical Considerations

The study protocol was approved by the Institutional Review Board (IRB) of the Seoul National University Dental Hospital (CRI26003). Because the study involved only fully deidentified historical dental chart images and no direct patient contact, informed consent was waived in accordance with the IRB guidelines. Voluntary informed consent was obtained from all participating dental students and clinicians prior to their involvement in the study. Data confidentiality and privacy were strictly maintained throughout the study.

## Results

### Overview

**Figure 3.**
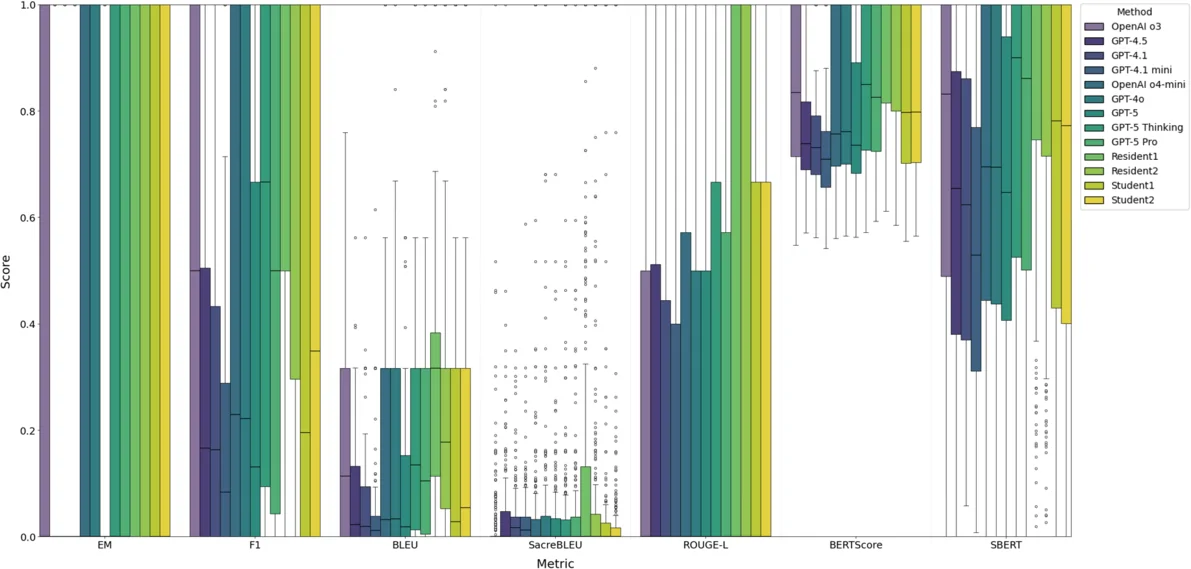
Comparison of evaluation scores across multiple metrics for ChatGPT models and human responses. Colored boxplots represent item-level scores for each model and human group; higher values indicate closer agreement with reference answers. BERT: bidirectional encoder representations from transformers; BLEU: bilingual evaluation understudy; EM: exact match; ROUGE-L: recall-oriented understudy for gisting evaluation-longest common subsequence; SBERT: sentence bidirectional encoder representations from transformers.

A total of 360 questions (15 charts×24 questions) were scored against the gold-standard answers. Model alignment, as measured by SBERT, generally exceeded that measured by string-based exactness metrics, indicating greater sensitivity to semantic adequacy than to exact matching. According to SBERT, the highest-performing model was GPT-5 Thinking, with a median score of 0.900 (IQR 0.525‐1.000), followed by GPT-5 Pro at 0.861 (IQR 0.501‐1.000) and OpenAI o3 at 0.831 (IQR 0.489‐1.000). Intermediate scores were observed for OpenAI o4-mini (0.695, IQR 0.444‐1.000) and GPT-4o (0.694, IQR 0.438‐1.000). Lower-performing models included GPT-4.5 (0.654, IQR 0.380‐0.874), GPT-5 (0.647, IQR 0.407‐0.940), GPT-4.1 (0.623, IQR 0.370‐0.861), and GPT-4.1 mini (0.529, IQR 0.311‐0.769). Within this framework, GPT-5 Thinking demonstrated the highest semantic alignment with reference answers, whereas GPT-4.1 mini exhibited the lowest. Full distributions across all 7 evaluation metrics are shown in Figure 3, with per-item scores available in Multimedia Appendix 3.

BLEU and SacreBLEU results reproduced the broad performance groupings identified by SBERT but revealed metric-specific reorderings among closely clustered models. ROUGE-L scores highlighted additional local inversions that differed from the SBERT rankings. These cross-metric differences emphasize that exactness-oriented and semantics-oriented metrics capture distinct types of model errors and should be interpreted jointly. In Figure 3, boxplots show that resident clinicians scored at or near the maximum for EM, *F*_*1*_-score, BERTScore, and SBERT, whereas the LLM and student groups formed lower clusters, with near-zero medians for EM, SacreBLEU, and ROUGE-L.

**Figure 4.**
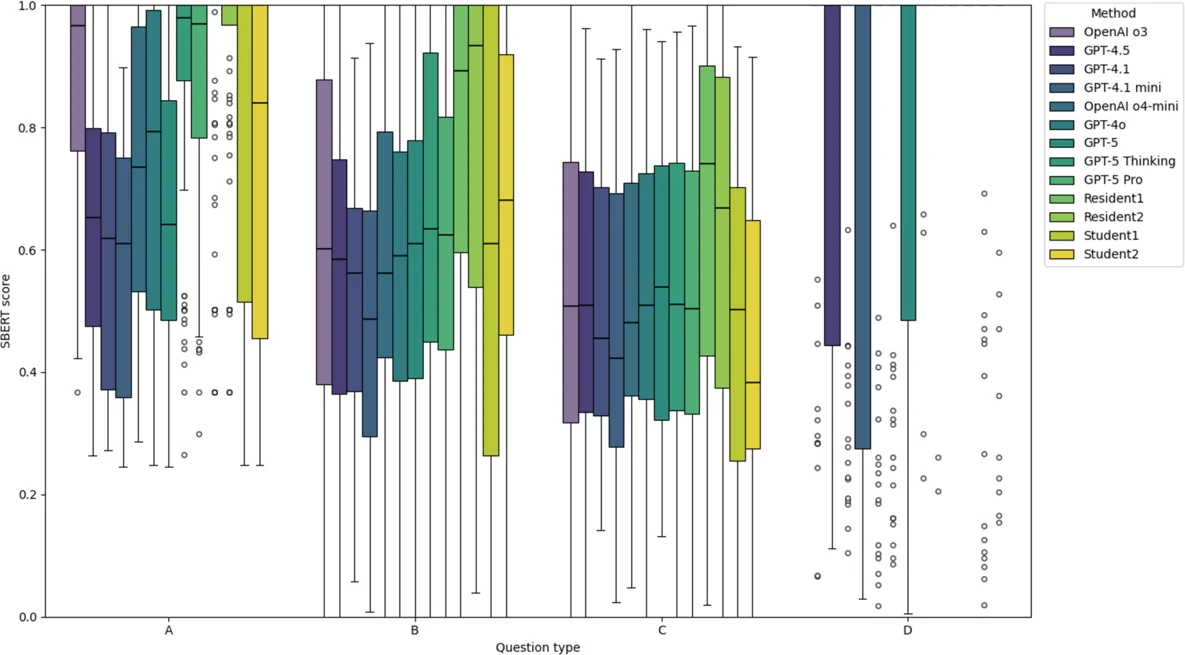
Boxplots of sentence bidirectional encoder representations from transformers (SBERT) scores by question type across ChatGPT models. Colored boxplots show item-level SBERT similarity scores for each model and human responses across question Types A-D (A, general; B, dental; C, interpretation; D, absent information); higher scores indicate closer semantic alignment with reference answers. Circles represent outliers. SBERT: sentence bidirectional encoder representations from transformers.

Stratification by the 4 predefined question types (90 items per type) revealed task-dependent variability in model performance. Figure 4 presents SBERT boxplots by model and question type. GPT-5 Thinking, GPT-5 Pro, and resident clinicians demonstrated higher and narrower score distributions for Types A and B, indicating consistent performance on explicit chart facts and tooth- or procedure-specific items. Type C (interpretation requiring multientry synthesis) showed more overlapping distributions across models, reflecting greater difficulty and variability. For Type D (absent information and abstention control), most groups achieved relatively high scores, indicating effective recognition of missing information. The SBERT-based relative rankings (highest to lowest) for each type were as follows.

- Type A, general (explicit chart facts): GPT-5 Thinking, GPT-5 Pro, OpenAI o3, GPT-4o, OpenAI o4-mini, GPT-4.5, GPT-5, GPT-4.1, and GPT-4.1 mini
- Type B, dental (tooth/procedure-specific): GPT-5 Thinking, GPT-5 Pro, GPT-5, OpenAI o3, GPT-4o, GPT-4.5, GPT-4.1, OpenAI o4-mini, and GPT-4.1 mini
- Type C, interpretation (multientry synthesis): GPT-5, GPT-5 Thinking, GPT-4.5, GPT-4o, OpenAI o3, GPT-5 Pro, OpenAI o4-mini, GPT-4.1, and GPT-4.1 mini
- Type D: absent information (abstention control): GPT-5 Pro, GPT-5 Thinking, OpenAI o3, GPT-4.1, GPT-4o, OpenAI o4-mini, GPT-4.5, GPT-5, and GPT-4.1 mini

For Types A, B, and D questions, the rank order generally mirrored the overall model analysis. Type A (fact retrieval) demonstrated high agreement with the reference answers; common factual items frequently achieved EM=1, reflecting reliable extraction of explicitly documented fields. In contrast, Type C (interpretation) produced compressed SBERT distributions, with medians ranging from 0.423 (IQR 0.278-0.692; GPT-4.1 mini) to 0.539 (IQR 0.321-0.738; GPT-5), a range of only 0.116 across models, and low EM rates, indicating that no model demonstrated a practically distinct advantage in multientry synthesis. Responses varied in evidence focus (eg, treatment plan, procedure note, or progress entry) and granularity (tooth-level or patient-level), resulting in heterogeneous rationales even when semantic similarity scores were closely clustered. For Type D, multiple models shared identical median scores; therefore, mean SBERT values were used to differentiate the rankings.

Illustrative examples are provided in Table 3 and Table 4, showing a convergent Type B item with uniformly correct factual outputs and a divergent Type C item with heterogeneous rationales and reduced semantic similarity.

**Table 3.** Example Type B (tooth-specific) question on the percussion finding of tooth number 36 at the initial visit, showing largely convergent factual answers and corresponding sentence bidirectional encoder representations from transformers (SBERT) similarity scores for human raters and ChatGPT models. SBERT similarity scores range from 0 to 1, with higher values indicating closer semantic agreement with the gold-standard answer. Question: What was the percussion finding on tooth number 36 at the initial visit?

Model	Answer	SBERT similarity score (range 0-1)
Gold Answer	per^a^ (−)	1.000
Resident 1	The tooth shows a negative percussion finding; per (−)	0.652
Resident 2	At the initial visit, tooth number 36 showed a negative percussion response	0.187
Student 1	Negative	0.143
Student 2	(–), no response to percussion	0.402
OpenAI o3	Negative percussion response	0.177
GPT-4.5	Percussion test positive (Pal^b^+, distal), bite test negative	0.035
GPT-4.1	At the initial visit, tooth number 36 showed percussion (–), occlusal (–), palatal (+), and bite (–, bite stick)	0.283
GPT-4.1 mini	At the initial visit, tooth number 36 showed per (−), bite (−), pal (+), and distal sinus tract (+)	0.265
OpenAI o4-mini	At the initial examination, tooth number 36 showed a negative response to percussion; per (−)	0.416
GPT-4o	At the initial visit, tooth number 36 showed a positive percussion response on palpation	0.294
GPT-5	At the initial visit, the percussion finding of tooth number 36 was pain on occlusion (−), bite (−), and pain on bite stick (−)	0.187
GPT-5 Thinking	Per (–)	0.569
GPT-5 Pro	Per (–)	0.550

a per: percussion.

b pal: palpation.

**Table 4.** Example Type C (interpretation) question on planned treatment for teeth numbers 37 and 47, illustrating divergent rationales and sentence bidirectional encoder representations from transformers (SBERT) similarity scores across human raters and ChatGPT models. SBERT similarity scores range from 0 to 1, with higher values indicating closer semantic agreement with the gold answer. Question: If treatment is performed on teeth numbers 37^a^ and 47^b^, what kind of treatment would be done?

Model	Answer	SBERT similarity score (range 0-1)
Gold answer	After performing a CLP^c^ by trimming the gum to increase the clinical crown length, caries treatment should be carried out, with RCT^d^ if necessary.	1.000
Resident 1	After performing CLP which trims the gum to increase the clinical crown length, caries treatment should be conducted with a possibility of RCT.	0.950
Resident 2	If necessary, caries treatment should be performed; however, CLP must be carried out beforehand, and there is a high likelihood of requiring RCT during the course of treatment.	0.942
Student 1	Distocervical caries can be removed and restored with resin.	0.242
Student 2	A gingivectomy or CLP may be required.	0.380
OpenAI o3	After a subgingival crown lengthening procedure, caries removal and conservative/restorative treatment, including root canal treatment if necessary, are required.	0.600
GPT-4.5	If treatment is performed on teeth numbers 37 and 47, there is a high likelihood that RCT will be required.	0.576
GPT-4.1	If treatment is performed on teeth numbers 37 and 47, regular monitoring of subgingival caries and, if necessary, conservative restorative treatment and root canal therapy will be considered.	0.206
GPT-4.1 mini	If treatment is performed on teeth numbers 37 and 47, a re-evaluation will be conducted after one year, with instructions on the use of interdental brushes and molar brushes, along with regular check-ups and additional treatment if necessary.	0.459
OpenAI o4-mini	If treatment is performed on teeth numbers 37 and 47, follow-up with bitewing X-rays at 6-month intervals is recommended. If symptoms or progression are observed, conservative restorative treatment or, if necessary, root canal therapy should be considered. It was also explained that careful case selection, including consideration of CLP, is required during treatment.	0.441
GPT-4o	It is explained that CLP is required beforehand, and the possibility of RCT is high.	0.805
GPT-5	High likelihood of RCT following CLP.	0.864
GPT-5 Thinking	After CLP, caries removal and restoration will be performed, with RCT if necessary.	0.888
GPT-5 Pro	Crown lengthening, followed by caries removal and restoration, with RCT if necessary.	0.548

a 37: left mandibular second molar.

b 47: right mandibular second molar.

c CLP: crown-lengthening procedure.

d RCT: root canal treatment.

### Human-Model Comparison

**Figure 5.**
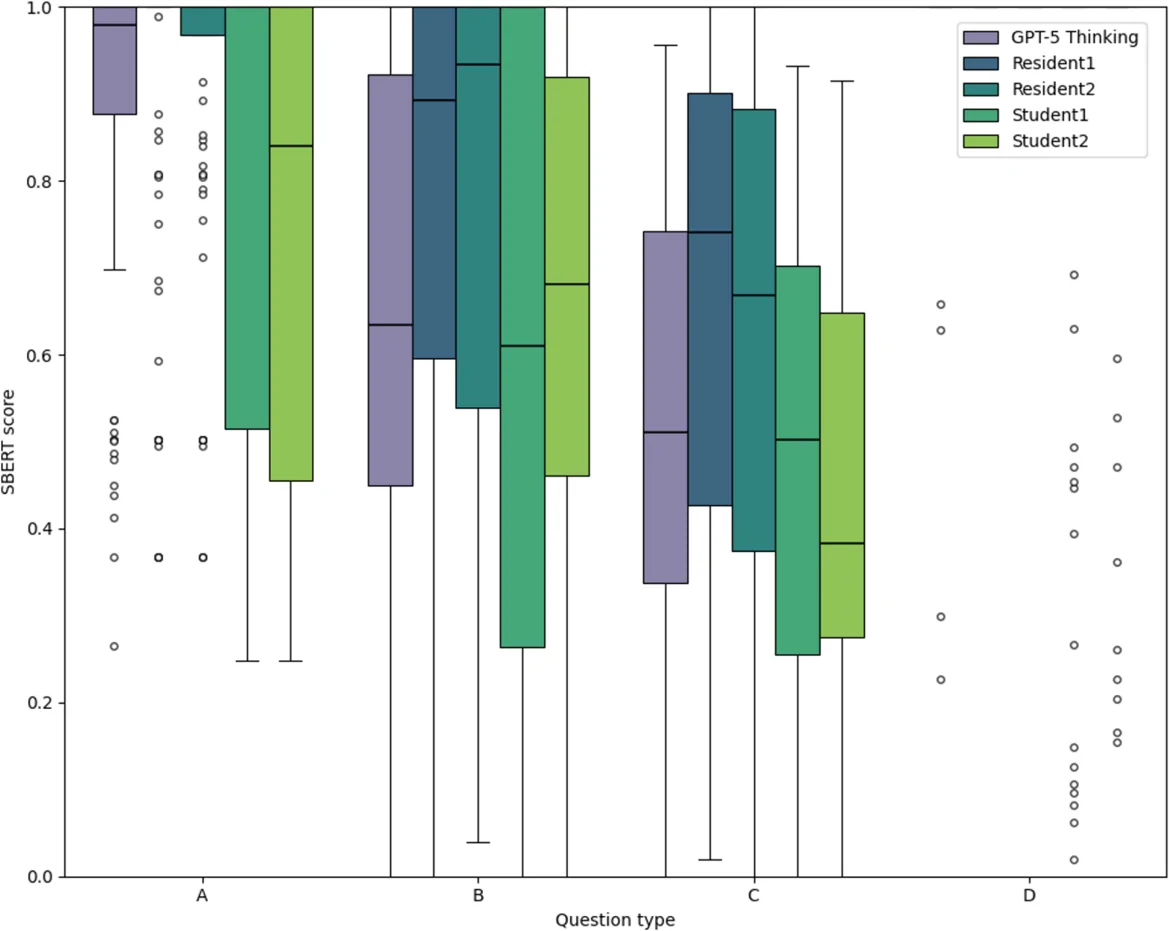
Boxplots of sentence bidirectional encoder representations from transformers (SBERT) scores by question type for GPT-5 Thinking and human responses. Colored boxplots show item-level SBERT scores for GPT-5 Thinking, Resident 1 and Resident 2, and Student 1 and Student 2 (third-year dental students). SBERT: sentence bidirectional encoder representations from transformers.

The descriptive statistics (median and IQR) for each question type are summarized in Table 5, and the corresponding pairwise comparisons with GPT-5 Thinking, with effect sizes, are provided in Table S2 in Multimedia Appendix 2. These comparisons are based on SBERT similarity to the gold standard, which reflects semantic adequacy rather than verified clinical correctness. Embedding similarity can rate clinically opposite findings as similar and clinically equivalent but differently worded answers as dissimilar. These contrasts, therefore, should be read as semantic proximity to the reference and interpreted alongside the content-based clinical accuracy analysis reported below. SBERT scores against the gold-standard answers were compared between GPT-5 Thinking and 4 human baselines—Student 1 and Student 2 (preclinical-to-clinical transition students) and Resident 1 and Resident 2 (first-year residents)—across question Types A-D (n=360), as shown in Figure 5. Because the distributions were nonnormal with unequal variances, Kruskal-Wallis tests indicated significant overall group differences for all 4 question types (all *P*<.001; ε²=0.052‐0.091). Dunn post hoc tests with Holm adjustment showed that Student 1 did not differ significantly from GPT-5 Thinking on Type A (*P*≥.99), Type B (*P*≥.99), Type C (*P*=.39), or Type D (*P*=.16). Student 2 differed only on Type D, scoring lower than GPT-5 Thinking (*P*=.002; δ=−0.27; 95% CI −0.41 to −0.13). Among resident clinician groups, Resident 1 scored higher than GPT-5 Thinking on Types A (*P*=.01; *δ*=0.29; 95% CI 0.13-0.44) and C (*P*=.04; *δ*=0.25; 95% CI 0.08-0.41) but did not differ significantly on Type B (*P*=.07) and scored lower on Type D (*P*<.001; δ=−0.35; 95% CI −0.49 to –0.21). Resident 2 scored higher than GPT-5 Thinking on Types A (*P*=.03; *δ*=0.25; 95% CI 0.09-0.41), B (*P*=.02; *δ*=0.28; 95% CI 0.11-0.44), and C (*P*=.04; *δ*=0.26; 95% CI 0.09-0.42), with no significant difference on Type D (*P*=.23). Overall, effect sizes were negligible to small except for the Resident 1 Type D contrast, which was medium in magnitude. These results indicate that GPT-5 Thinking was broadly comparable to the students across most tasks and remained below resident-level performance for several Types A-C contrasts. Type D warrants particular caution. Because all groups shared identical ceiling medians and IQRs of 1.000, the 2 significant contrasts arose only from a few lower-scoring tail items, reflecting distributional subtleties rather than a real difference in recognizing absent information.

**Table 5.** Comparison of sentence bidirectional encoder representations from transformers (SBERT) similarity to the gold-standard answers between GPT-5 Thinking and human raters, by question type.

Question type	GPT-5 Thinking	Resident 1	Resident 2	Student 1	Student 2
SBERT similarity to gold standard, median (IQR)					
Type A (general)	0.978 (0.870‐1.000)	1.000 (1.000‐1.000)	1.000 (0.978‐1.000)	0.994 (0.863‐1.000)	0.904 (0.744‐1.000)
Type B (dental)	0.813 (0.525‐0.969)	0.968 (0.616‐1.000)	0.991 (0.649‐1.000)	0.642 (0.336‐1.000)	0.744 (0.404‐1.000)
Type C (interpretation)	0.556 (0.371‐0.650)	0.641 (0.478‐0.861)	0.688 (0.413‐0.820)	0.478 (0.221‐0.607)	0.406 (0.271‐0.580)
Type D (absent information)	1.000 (1.000‐1.000)	1.000 (1.000‐1.000)	1.000 (1.000‐1.000)	1.000 (1.000‐1.000)	1.000 (1.000‐1.000)
Dunn post hoc comparison versus GPT-5 Thinking (*P* value)					
Type A (general)	—^a^	.01	.03	>.99	.37
Type B (dental)	—	.07	.02	>.99	>.99
Type C (interpretation)	—	.04	.04	.39	.13
Type D (absent information)	—	<.001	.23	.16	.002

a Not applicable.

### Reliability of the Gold Standard

The independent senior specialist judged all 360 gold-standard answers to be clinically acceptable (100% endorsement). Among the clinical reference raters, agreement on the extracted clinical value was very high (Gwet AC1=0.99 overall and ≥0.98 for every task type; Table S3 in Multimedia Appendix 2), indicating that the gold standard was reproducible across independent, qualified clinicians. The canonical-value extraction agreed with an independent dentist on 97.2% of the validation subset. When the 2 third-year students were included, agreement was lower (Gwet AC1=0.83; Table S4 in Multimedia Appendix 2), reflecting their developing chart-reading competence rather than the unreliability of the reference. SBERT cosine and BERTScore *F*_*1*_-score showed the same ordering at lower magnitudes (clinical reference raters ≈ 0.85 and 0.87) and are reported only as descriptive checks.

### Robustness of the Model Ranking and Safety

To confirm that the ranking reflected clinical content rather than embedding similarity alone, all 9 models were independently rescored for clinical correctness against the gold-standard value. This content-based clinical-accuracy ranking was closely aligned with the SBERT ranking (Spearman ρ=0.90; *P*=.001; Table S5 in Multimedia Appendix 2), with GPT-5 Pro, GPT-5 Thinking, and OpenAI o3 forming the leading group (clinical accuracy 96.4%, 95.6%, and 91.4%, respectively) and GPT-4.1 mini the lowest (68.9%). This convergence indicates that the model ordering was not an artifact of semantic-similarity scoring. On the absent-information (Type D) task, the proportion of fabricated answers in Table S6 in Multimedia Appendix 2, supplying a value the chart did not contain instead of abstaining, rose as model capability fell, from 2.2% (GPT-5 Pro) and 4.4% (GPT-5 Thinking) to 28.9% (GPT-5) and 46.7% (GPT-4.1 mini), whereas all 3 clinical raters abstained appropriately on every such item. This focused exploratory comparison, limited to the tooth- and procedure-specific task, showed no significant difference between Korean-script and English or numeric answers (87.8% vs 90.4%; *P*=.22), indicating that the mixed language input did not by itself drive performance differences within this task. Given its narrow scope, this result is descriptive and does not constitute a comprehensive multilingual evaluation.

### Expert Adjudication of Clinically Consequential Discrepancies

The exact agreement between the 2 adjudicators was 91.5% (n=59; Gwet AC1=0.879; linearly weighted κ=0.89), and all 5 disagreements were between adjacent categories and were resolved by consensus. Of the 59 adjudicated responses, 10 were rated as clinically consequential: 4 of 23 were content-incorrect responses, and all 6 were Type D fabrications. Within the adjudicated subset, clinically consequential ratings occurred only among responses previously classified as content-incorrect or fabricated. The 10 identified cases represented 1.4% of the 720 outputs generated by the 2 models; however, because adjudication was subset-based, this percentage should not be interpreted as a full-sample incidence estimate. None of the 30 sampled low-SBERT responses previously classified as content-correct was rated as clinically consequential, indicating that low SBERT similarity in this sampled stratum reflected wording differences rather than clinical error, as shown in Table S7 in Multimedia Appendix 2.

### Exploratory Qualitative Feedback

Content analysis of the open-ended responses from the 5 participants yielded a small set of recurrent themes for each question, summarized with representative excerpts in Table S1 in Multimedia Appendix 2. For perceived advantages, time efficiency was noted by all 5 participants (n=5), followed by accessibility, immediate feedback, repeated learning, and support for self-directed learning (each n=3). For perceived disadvantages, the possibility of AI errors or inaccuracies was raised by all participants (n=5), with the lack of clinical context, overreliance associated with reduced critical thinking, and privacy or data-leakage risks each noted by 2 participants (n=2). In comparing the tool with traditional faculty-guided instruction, most participants (n=4) viewed AI as a supplementary tool to be used alongside faculty rather than as a replacement, and 3 participants (n=3) considered it particularly useful during the early learning stages for terminology and basic chart reading. Notably, 4 participants (n=4) emphasized that faculty expertise remains necessary when contextualized clinical reasoning or treatment decisions are required, indicating that participants regarded AI as most appropriate for foundational, evidence-locatable tasks while reserving complex clinical judgment for faculty guidance.

## Discussion

### Principal Results

In this image-based benchmark of dental chart interpretation, the main findings were 3-fold. First, reasoning-optimized models led across tasks: GPT-5 Thinking achieved the highest overall semantic alignment with the gold standard (SBERT median 0.900), followed by GPT-5 Pro and OpenAI o3, while the smallest models performed worst, and a content-based clinical-accuracy re-scoring reproduced this ranking (Spearman ρ=0.90; *P*=.001). Second, in the human comparison, GPT-5 Thinking was statistically indistinguishable from dental students on most question-type contrasts but remained below first-year residents on several factual, dental-specific, and interpretive (Types A-C) contrasts, with multientry synthesis (Type C) being the most challenging task across groups. Third, abstention on absent-information (Type D) items was generally reliable, although fabrication rose sharply as model capability fell (from 2.2% for GPT-5 Pro to 46.7% for GPT-4.1 mini), whereas all clinical raters abstained appropriately on every such item. Together, these results position the strongest multimodal models as potential supervised aids for chart-reading practice rather than as substitutes for clinical expertise.

Each question paired a dental chart image with a text prompt, requiring models to parse chart layouts, interpret symbolic markings, and align visual cues with textual questions, similar to emerging evaluations of medical multimodal LLMs in radiology and nuclear medicine, where vision–language integration and domain grounding are critical determinants of performance [30-33]. Consistent with reviews noting that LLM outcomes are strongly task- and modality-dependent and that safety and hallucination control are key concerns in clinical settings [34-38], the heterogeneous patterns across models and task types are compatible with differences in reasoning architecture, safety alignment, and multimodal training described in GPT-5 system documentation [39,40].

Across Type A items (explicit chart-based facts), GPT-5 Thinking, GPT-5 Pro, and OpenAI o3 formed the leading group. For Type B items (tooth- and procedure-specific reasoning), GPT-5 Thinking and GPT-5 Pro again ranked highest, followed by GPT-5. Public documentation describes GPT-5 Thinking as a reasoning-focused variant trained with reinforcement-learning methods and “safe completions” designed to reduce unsafe or overconfident outputs and GPT-5 Pro as a configuration with additional test-time compute [39,40]. These design characteristics align with the relatively stable factual performance observed, though other factors, such as visual processing pipelines and context handling, may also contribute [30-33]. For Type C items, which required synthesis across multiple chart regions and textual entries, GPT-5 outperformed GPT-5 Thinking and GPT-5 Pro. One possible interpretation is that general-purpose tuning and strong long-context capabilities facilitated flexible integration of disparate findings [39,40], whereas the more cautious, stepwise reasoning of GPT-5 Thinking and GPT-5 Pro may have limited the breadth of synthesized interpretations. However, this post hoc explanation cannot be definitively separated from potential influences of context-window use, input batching, or image-handling behavior. Type D items assessed abstention behavior in the presence of missing information. GPT-5 Pro demonstrated the most reliable abstention performance, followed by GPT-5 Thinking and OpenAI o3, whereas GPT-5 performed comparatively poorly. Reasoning-focused models such as GPT-5 Thinking are reported to prioritize safety and adherence to instructions through reinforcement learning and safe completions [39]. The observed pattern is consistent with more conservative responses and aligns with prior work on abstention and hallucination control in safety-tuned models [41-45]; however, this does not constitute direct evidence that specific training procedures caused the observed behavior.

Aggregated across task types, the overall ranking was GPT-5 Thinking, GPT-5 Pro, OpenAI o3, OpenAI o4-mini, GPT-4o, GPT-4.5, GPT-5, GPT-4.1, and GPT-4.1 mini. The leading positions of GPT-5 Thinking and GPT-5 Pro in this multimodal dental setting suggest that, across a mixture of factual, procedural, integrative, and abstention-focused tasks, cautious structured reasoning and calibrated uncertainty may contribute more to overall utility than maximally flexible generation. The strong performance of OpenAI o3 is consistent with its role as a reasoning-focused predecessor [39]. The relatively high placement of OpenAI o4-mini indicates that some questions—particularly those requiring explicit factual recognition or simple abstention—can be addressed effectively by more parameter-efficient models, consistent with findings in medical multimodal imaging [30-33]. Between-model differences may, however, reflect variation in context-window size, segmented uploads of large charts, differences in OCR and image-processing pipelines, and heterogeneous handling of Korean-language inputs, cautioning against strong claims about intrinsic capability. These findings tentatively support a model-by-task strategy, to be validated prospectively. Within the demanding Type B task, Korean-script and English or numeric answers did not differ (87.8% vs 90.4%; *P*=.22), indicating that code-mixed input did not by itself drive the results.

Against human raters, GPT-5 Thinking was broadly comparable to students and below first-year residents on several extraction and interpretive contrasts. Most student-model comparisons were not statistically significant, although Student 2 scored lower on the absent-information task. Practically, for basic extraction and description of information, GPT-5 Thinking matched students transitioning to clinical training, while remaining below residents on several Type A-C contrasts, consistent with prior education evaluations in which ChatGPT-level systems often reach, but rarely surpass, student performance on written examinations and vignette-style assessments [38,46]. This pattern was clearest on the more “dentally demanding” tasks—Type B (tooth- and procedure-specific reasoning) and Type C (multientry synthesis)—where resident clinicians generally had higher scores than GPT-5 Thinking and students, suggesting differences in prioritization and contextual interpretation. On Type D, all groups clustered near the high end of the score range, frequently recognizing missing information and avoiding overinterpretation, and reached identical ceiling medians. Where pairwise contrasts nonetheless reached significance, the differences stemmed from a few tail items rather than from a systematic gap in abstention and are best read as distributional rather than clinically meaningful. Although GPT-5-family models are described as incorporating approaches intended to reduce hallucination and promote conservative responses under uncertainty [37,39,44], this finding provides reassurance that the model often adopts a safe abstention strategy when key information is missing, though it does not imply parity with clinicians. Quantitatively, the proportion of fabricated answers on absent-information items ranged from 2.2% (GPT-5 Pro) and 4.4% (GPT-5 Thinking) to 28.9% (GPT-5) and 46.7% (GPT-4.1 mini), whereas all 3 clinical raters abstained appropriately on every item, underscoring that the cheapest models are unsafe for unsupervised use. Overall, GPT-5 Thinking is most appropriately framed as a supervised support tool or virtual peer learner, not a substitute for clinician judgment [34,35,37].

The 7 metrics captured different aspects of model behavior. Exactness-oriented metrics (EM, ROUGE-L, and SacreBLEU) were largely uninformative, reaching nonzero medians essentially only for residents because they reward verbatim reproduction rarely expected in open-ended explanations. Lexical-overlap metrics (*F*_*1*_-score, BLEU) gave a clearer but still surface-level hierarchy (residents >GPT-5 Thinking >OpenAI o3 ≈ GPT-5 Pro >remaining models and students). Semantic metrics were most informative: on SBERT, resident clinicians scored 1.0, GPT-5 Thinking 0.900, GPT-5 Pro 0.861, and OpenAI o3 0.831, with students around 0.77‐0.78 and other models lower. Because these embedding metrics track sentence-level meaning rather than wording, GPT-5 Thinking, GPT-5 Pro, and OpenAI o3 constitute a “reasoning-optimized” cluster that is semantically closer to resident clinician answers than both students and earlier models. Within the GPT-5 family, the gap between GPT-5 and GPT-5 Thinking further implies that architectural and alignment choices aimed at deeper reasoning translate into measurable gains in clinical content quality, beyond what can be achieved by scale alone [41-44]. Median scores for GPT-5 Thinking fell between students and residents; wide IQRs reflect varying difficulty of the clinical questions, the probabilistic nature of LLM responses, and the individual differences in clinical experience among the students. Importantly, embedding similarity reflects semantic adequacy rather than clinical correctness: it can rate clinically opposite findings as highly similar (eg, per [+] vs per [−]) and clinically equivalent statements as dissimilar when their wording differs. SBERT is therefore best interpreted as a scalable proxy for semantic proximity to the reference, not as a direct measure of clinical correctness, and all SBERT-based human-model contrasts should be interpreted accordingly. To guard against this limitation, the SBERT ranking was corroborated by an independent content-based clinical-accuracy rescoring of all 9 models, which reproduced the same ordering (Spearman ρ=0.90; *P*=.001); this content-based analysis, rather than embedding similarity alone, anchors the interpretation of relative model performance. Results should still be read cautiously given the modest, single-institution sample.

The expert-adjudicated subset analysis reinforces this reading of the automated metrics. Within the adjudicated subset, clinically consequential ratings occurred only among responses previously flagged as content-incorrect or fabricated, and none occurred in the sampled low-similarity responses previously classified as content-correct. The 10 identified cases represent 1.4% of the 720 outputs generated by the 2 models; however, because adjudication was subset-based, this percentage should not be interpreted as a full-sample incidence estimate. Because adjudication was limited to the 2 best-performing models and used a rubric-based consequence scale rather than observed educational outcomes, these findings characterize the adjudicated subset within this benchmark rather than clinical performance.

### Implications for Dental Education

From an educational standpoint, the findings suggest that GPT-5 Thinking could potentially serve as a supervised reference and secondary-reader tool within chart-reading training. It is particularly suited for exercises emphasizing evidence identification, omission detection, and uncertainty recognition, skills central to safe documentation and clinical reasoning. Given the safety risks associated with unsupervised LLM errors in direct patient care, focusing on chart interpretation rather than definitive diagnosis better reflects the potential utility of LLMs as safe cognitive training tools in risk-free educational settings. Because the model matched novice learners on most contrasts but stayed below residents on several Type A-C tasks (notably Type B and C), human supervision remains essential. Rather than replacing instructor guidance, multimodal LLMs can facilitate guided education, allowing students to query chart content, test hypotheses, and receive structured feedback under supervision. This perspective aligns with automation-bias literature emphasizing verification and guardrails [47] and with the findings by Hattie and Timperley [48] that high-quality feedback is an important influence on achievement, suggesting that multimodal LLMs may be most beneficial when integrated into feedback-rich, supervised learning activities.

Practical integration into dental curricula can be achieved through digital exercises using deidentified charts, case-based seminars, and self-directed study tasks. These activities should emphasize locating relevant entries, reconstructing treatment timelines, and verifying documented procedures, thereby helping students develop both chart-reading literacy and AI literacy. Advances in multimodal and interactive interfaces further align training with clinical practice, allowing students to upload chart images, use voice input during simulations, and receive immediate feedback [49].

Safe and responsible adoption requires institutional policies that define permissible uses, mandate verification against original charts, and record model details for assignments. The use of deidentified data and audit logs should be standard to ensure transparency and accountability. Within these guardrails, multimodal LLMs can function as collaborative aids in guided education, promoting deliberate practice and structured feedback while maintaining professional oversight. This approach aligns with Masters’ recommendations for ethical AI use in health professions education, which emphasize governance, transparency, and accountability frameworks [50].

An exploratory qualitative feedback analysis obtained from study participants and summarized in Table S1 in Multimedia Appendix 2 provided additional insight into the perceived educational usefulness of AI-assisted dental chart interpretation. Participants consistently identified time efficiency, accessibility, immediate feedback, and support for self-directed learning as key advantages of the tool. These findings are consistent with emerging evidence suggesting that generative AI can function as an on-demand educational resource that enhances learner autonomy and supports personalized learning in health professions education [51]. Prior studies have also suggested that LLMs may serve as valuable educational aids by providing rapid access to information, supporting formative feedback, and facilitating independent learning outside traditional instructional settings [8]. Furthermore, AI tools may function as cognitive scaffolds that help learners navigate complex clinical information while promoting active engagement with educational content and supporting the development of foundational competencies [51,52]. Notably, participants perceived the tool as useful in early clinical training, when learners are first exposed to unfamiliar terminology, chart structures, and clinical documentation. This finding suggests that multimodal AI may facilitate the preclinical-to-clinical transition by providing timely support and opportunities for deliberate practice.

Participants also expressed concerns about inaccuracies, overreliance, privacy, and the inability of AI systems to fully capture contextual reasoning. Importantly, both students and residents regarded AI as a supplementary educational tool rather than a replacement for faculty-guided instruction, particularly when complex clinical reasoning and judgment were required. These perceptions align with recent discussions in medical education emphasizing that generative AI should augment rather than replace human expertise and that learners must be trained to critically evaluate AI-generated outputs [53]. Concerns regarding cognitive dependency and reduced independent reasoning have likewise been highlighted as important challenges associated with the educational use of generative AI [52,53]. Together, these findings support multimodal AI as an adjunctive learning technology that enhances chart-reading literacy under supervision while preserving the central educational role of faculty.

### Comparison With Prior Work

This study makes 2 novel contributions. First, it represents the first systematic evaluation of multimodal LLMs for dental chart image interpretation. Second, it implements a multiaxis performance assessment across 4 distinct task types, including general fact retrieval (Type A), tooth- or procedure-level retrieval (Type B), interpretive and inference reasoning (Type C), and absent-information and abstention control (Type D).

Prior studies have primarily examined LLMs’ ability to extract information from text-based inputs, such as discharge summaries or narrative clinical notes. While these tasks assess factual comprehension, they omit the spatial information inherent in clinical records, particularly in dental charts that include odontograms, tooth-number grids, and procedure maps. Linearizing such records into text disrupts the contextual relationships between spatial elements and their annotations. Evaluating image-based chart inputs, therefore, probes multimodal reasoning under more realistic conditions, reflecting how clinicians interpret dental charts by integrating both spatial and textual cues. Although multimodal capability has been documented in several medical imaging domains, such as radiography and pathology slide analysis [30], comparable evaluations using dental chart images have not previously been reported, motivating the present image-based benchmarking.

With respect to the second contribution, most prior evaluations have relied on multiple-choice or short-answer formats, such as the United States Medical Licensing Examination (USMLE), reporting aggregate accuracy without decomposing performance by task type [8]. The present findings align with previous reports indicating that LLMs can reach or exceed passing thresholds on structured, well-specified examinations but remain less reliable on open-ended reasoning and fact-checking tasks [7,8]. Reviews have similarly emphasized hallucination and inconsistent abstention as persistent limitations, highlighting the need for human verification and explicit guardrails in both educational and clinical contexts [54,55]. In dental education, a mixed methods study of ChatGPT in undergraduate training reported benefits for rapid clarification, idea generation, and formative feedback, while also noting factual errors and over-reliance, prompting recommendations for supervised, source-verified use [15].

Language effects further contextualize the present results. Earlier work suggested higher LLM performance in English compared with other languages; however, recent multimodal models (eg, GPT-4o) have narrowed this gap, demonstrating improved correctness and responsiveness in Japanese across general and medical imaging tasks, including radiology [56]. In dentistry, a peer-reviewed evaluation of GPT-4o and Gemini Advanced on the Korean National Dental Licensing Examination documented strong accuracy and consistency on well-specified, evidence-locatable items [57]. Emerging assessments of GPT-5 models in Korean similarly report robust performance on structured, multimodal tasks, findings broadly consonant with the present study’s mixed Korean and English chart data. Collectively, these studies suggest that recent multimodal LLM families are reducing prior language penalties, particularly when queries are context-bounded and answers can be anchored to identifiable evidence. The present language comparison, however, was a focused exploratory subanalysis within a single task rather than a controlled multilingual evaluation, so it can only contextualize these findings and cannot establish language equivalence.

### Practical Guidance for Use

The highest-performing models in the study, such as GPT-5 Thinking and GPT-5 Pro, achieved the greatest accuracy but required longer response times. In time-sensitive educational contexts, such as self-directed chart-reading practice or preparation for case-based seminars, faster models like GPT-4o or OpenAI o4-mini may be more practical, as modest reductions in accuracy are offset by faster responses [58]. Two key implications emerge for educational use. First, model selection should be guided by task demands: faster models are suitable for routine information retrieval during practice, whereas reasoning-tuned models are preferable when accuracy and abstention are critical. Second, all outputs should be verified against the original record before clinical use, given the potential for errors when documentation is incomplete or absent [12,13]. Because the models were accessed through the consumer interface, exact compute cost and latency were not measured. Any cost-related guidance is therefore provisional and based on accuracy alone. On that basis, GPT-5 Thinking reached clinical accuracy close to the larger GPT-5 Pro, suggesting a more cost-effective option for educational deployment, whereas the smallest models, though inexpensive, abstained poorly and are not advisable for unsupervised use. These observations should be confirmed by a formal API-based benchmark of cost and latency alongside accuracy, which remains an important next step. For educators and learners considering practical use, the main conclusions can be summarized at a glance: across factual, dental-specific, interpretive, and abstention tasks, the strongest reasoning-optimized models (GPT-5 Thinking, GPT-5 Pro, and OpenAI o3) matched novice-student retrieval and abstained reliably, yet remained below resident-level interpretive reasoning, and fabrication of absent information rose steeply as model capability declined (from 2.2% to 46.7%). The practical guidance is therefore consistent across all 4 task types—multimodal LLMs are best used as supervised, verification-focused aids whose outputs are checked against the original record, rather than as autonomous interpreters. Although demonstrated here for dental records, this guidance parallels emerging questions about record-reading and documentation literacy in medical and nursing practice; whether the same supervised, verify-before-use framework transfers to other specialties or to adjacent health-profession settings remains an open question requiring external validation.

Although this study focused on educational benchmarking, one possible area for future investigation is whether multimodal LLMs could support clinical information retrieval from lengthy, mixed-language dental records. For example, identifying the date of a composite resin restoration on the upper right first premolar across multiple years of visits typically requires extensive manual review. A single structured query to an LLM can quickly surface the relevant note and date for confirmation, reducing search time and cognitive load. However, as this clinical-retrieval scenario was outside the scope of the present benchmark, it is best regarded as a direction for future prospective, safety-focused studies.

### Limitations

The dataset was drawn from a single department (Conservative Dentistry) from a single institution (SNUDH) and included only 15 charts. This study should therefore be regarded as an internal, single-center benchmark. Because multimodal chart interpretation depends heavily on local layout, documentation conventions, abbreviations, and specialty-specific workflows, which vary across institutions and electronic dental record systems, the single-center design carries an inherent risk of institutional bias, and the findings may not be directly generalizable to other specialties, institutions, record systems, or linguistic settings without external validation. Records contained both Korean and English entries, and results may differ in other language contexts.

The analysis reflected model versions selectable in the consumer interface under a Pro-tier subscription in early August 2025; model availability is both interface- and subscription-dependent and may differ at other times, under different tiers, or via API access. In addition, the models were accessed through the consumer interface, so exact token usage, latency, and compute cost were not measured.

Human comparisons were limited to 2 students and 2 residents, as their primary role was to establish a reference baseline for the AI models rather than to serve as a large-scale population sample. Nevertheless, this reduces the statistical precision of group contrasts and warrants confirmation in studies with larger human cohorts.

A further consideration concerns the independence of observations. Because the 24 questions for each patient were derived from the same chart, items from the same chart may share case-level characteristics such as complexity, documentation style, and image quality, so their scores are not fully independent. The nonparametric tests applied here treat items as independent and do not account for this within-chart clustering. The effective sample size is therefore smaller than the nominal number of items, and the item-level comparisons should be regarded as exploratory. Confirmatory analyses using clustered or mixed effects models with the chart as a grouping factor would help verify the robustness of these findings.

Reliability and scoring relied on semantic and content-based proxies rather than a formal clinical-harm rubric; although SBERT was triangulated with content-level agreement and canonical-value extraction was validated against an independent dentist on a 20% subset (97.2% agreement), residual error cannot be excluded. The expert-adjudicated subset analysis addressed clinically consequential discrepancies within this benchmark (Table S7 in Multimedia Appendix 2), but prospective grading of clinical harm under real educational use, by a larger expert panel, remains important future work.

In addition, the 360 questions were authored by a single clinical researcher (AYC) and were not independently reviewed for wording before evaluation, which may introduce authoring bias. This is most relevant for the interpretive Type C items, where multiple clinically acceptable framings and differing evidence-prioritization strategies can exist, potentially shaping item difficulty, ambiguity, and, in principle, model ranking. Several design features mitigate this concern—the identical question set was applied to all models, the gold-standard answers were independently judged clinically acceptable with high interrater agreement (Gwet AC1=0.99), and the SBERT-based ranking was reproduced by a content-based clinical-accuracy rescoring (Spearman ρ=0.90)—but confirmation with independently reviewed or multiauthor question sets remains warranted.

Several methodological constraints also warrant caution. First, because evaluation was end-to-end, OCR accuracy was not independently measured, and the chart resolution (717×742 pixels), the maximum obtainable resolution from the EMR interface, may have constrained visual parsing; this nonetheless reflects the native, constrained outputs that students and clinicians actually use, making robustness to such inputs relevant to real-world utility. Second, evaluations used the consumer graphical user interface (GUI) rather than the API, and the study was not preregistered, as it was an exploratory benchmark. This reflects a deliberate trade-off between ecological validity and computational reproducibility [59]. The GUI captures how students and clinicians actually interact with these models, but it introduces GUI-specific uncontrolled variables such as proprietary system prompts, memory-state handling, rate limiting, and dynamic model routing that are not under experimental control. These factors are further amplified for the larger charts, whose images exceeded the 10-image message limit and were therefore uploaded in consecutive batches within a single conversation thread. Consequently, the present results should be interpreted as real-world interface benchmarking rather than fully controlled model benchmarking. Because the behavior of the same commercial LLM service has been shown to shift substantially over short periods as providers update it without public notice [60], the observed model rankings may not be fully stable under future platform updates or under API-based, version-pinned evaluation. Finally, as a comparative study with students, this study cannot demonstrate whether using these models improves learning outcomes. Future work should use larger, multi-institutional, multispecialty, and ideally multinational datasets, complement this ecological benchmark with API-based, version-pinned, preregistered evaluations, and incorporate formal clinical grading rubrics to assess educational impact, safety, response time, and efficiency under real clinical conditions.

### Conclusions

This study compared multimodal ChatGPT models with dental students and first-year residents in reading and interpreting dental chart images. GPT-5 Thinking demonstrated the highest overall SBERT performance and was broadly comparable to dental students, with no significant difference from Student 1 across Types A-D and only one significant contrast with Student 2 on Type D. Compared with residents, however, it remained lower on several Type A-C contrasts, particularly Type C and one Type B comparison; Type D findings should be interpreted cautiously because all groups had ceiling medians. These findings indicate that current multimodal models can approach student-level comprehension in structured chart reading but remain below resident-level reasoning in several resident comparisons. In educational settings, such models may serve as supervised support tools for practicing chart interpretation and verification. These findings derive from an internal, single-center benchmark, and external validation across multiple institutions, dental specialties, record systems, and language settings is required before broader claims can be made. As multimodal capabilities continue to advance, carefully integrated AI systems may have the potential to support early clinical training through guided education, offering scalable, feedback-driven support to strengthen chart-reading literacy.

## Supplementary material

10.2196/91809 Multimedia Appendix 1 Dataset of 360 case-specific questions (in both the original Korean and translated English), human reference answers, raw outputs from the 9 evaluated models, and direct URL links to the original conversational logs.

10.2196/91809 Multimedia Appendix 2 Supplementary tables including exploratory qualitative feedback on educational utility, interrater reliability of the human reference standard, robustness of the evaluation metrics, model safety (fabrication rates), effect sizes for human-model comparisons, and expert adjudication of clinically consequential discrepancies.

10.2196/91809 Multimedia Appendix 3 Complete per-item metric evaluation results across 360 questions, comparing each response with the gold-standard references using 7 metrics (Exact Match, *F*_1_-score, BLEU, SacreBLEU, ROUGE-L, BERTScore, and SBERT). The dataset includes results for all evaluated models as well as student and resident raters.

10.2196/91809 Checklist 1 STROBE checklist.

## Abbreviations

BERT: bidirectional encoder representations from transformers
BLEU: bilingual evaluation understudy
EM: exact match
EMR: electronic medical record
GUI: graphical user interface
IRB: Institutional Review Board
LLM: large language model
NLP: natural language processing
OCR: optical character recognition
ROUGE-L: recall-oriented understudy for gisting evaluation-longest common subsequence
SBERT: sentence bidirectional encoder representations from transformers
SNUDH: Seoul National University Dental Hospital
STROBE: Strengthening the Reporting of Observational Studies in Epidemiology
USMLE: United States Medical Licensing Examination
