# Supplementary material for: Multimodal Large Language Models for Dental Chart Image Interpretation: Cross-Sectional Benchmarking Study With Students and Clinicians
Source: JMIR Med Educ. 2026 Sep 4;12:e91809. doi: 10.2196/91809 (PMC13544698; doi:10.2196/91809)
Supplement: Supplementary file 1 [file mededu-v12-e91809-s002.docx]

**Table S1.** Qualitative feedback on the educational usefulness of AI-assisted dental chart interpretation from students, residents, and faculty (n=5)

| **Theme** | **Values, n (%)** | **Description** | **Excerpts from written responses** |
| --- | --- | --- | --- |
| **Question 1. Perceived advantages of AI-assisted dental chart interpretation** | | | |
| Time efficiency | 5 (100) | Quick confirmation of abbreviations and diagnoses; saves study time. | • “It used to take a long time to look up abbreviations on my own; AI lets me check quickly.”  • “Students get chart-analysis results instantly and can experience more cases.” |
| Accessibility (anytime, anywhere) | 3 (60) | Information obtained without time or place constraints, even without an instructor. | • “AI is faster and easy to access from anywhere.” |
| Immediate feedback | 3 (60) | Learners confirm whether their interpretation is correct and learn the reasons for errors. | • “I can immediately check whether my interpretation is correct.”  • “Immediate feedback lets you acquire knowledge and resolve questions.” |
| Repeated learning | 3 (60) | Weak points can be gathered and reviewed repeatedly. | • “You can collect the parts you understand poorly and check them several times.” |
| Self-directed learning | 3 (60) | Independent study with less reliance on an instructor. | • “You can ask and answer on your own, even without an educator present.” |
| **Question 2. Perceived disadvantages or concerns of AI-assisted dental chart interpretation** | | | |
| Possibility of AI errors / inaccuracy | 5 (100) | AI may give incorrect or inaccurate interpretations, causing confusion or flawed reasoning. | • “AI does not always interpret charts accurately, so accepting it as-is can cause confusion.”  • “Wrong information may form incorrect diagnostic thinking.” |
| Lack of clinical context | 2 (40) | AI may not reflect systemic status, behavior, or social background considered in real practice. | • “AI cannot fully reflect the patient's overall condition and clinical context.” |
| Over-reliance / reduced critical thinking | 2 (40) | Depending on AI answers may weaken critical thinking and independent clinical judgment. | • “Relying on AI rather than reasoning independently can lower critical thinking ability.” |
| Privacy / data leakage | 2 (40) | Risk of leaking patient information if personal data is accidentally included. | • “If a learner mistakenly includes personal information, there is a risk of data leakage.” |
| **Question 3. Comparison with traditional faculty-guided chart-reading instruction** | | | |
| Supplementary tool / used with faculty | 4 (80) | AI complements rather than replaces faculty; basic practice via AI, difficult judgment with faculty. | • “It is appropriate to use AI as a supportive tool, not a replacement.”  • “AI and faculty education should be used together.” |
| Faculty expertise for complex clinical judgment | 4 (80) | Faculty guidance remains necessary when contextualized clinical reasoning or treatment decisions are required. | • “For areas requiring clinical experience, faculty can be more accurate, so AI and faculty teaching should be used together.” |
| Useful in early learning stages | 3 (60) | Effective in the pre-clinical phase for terminology and basic chart reading. | • “In the early stage, even reading charts and abbreviations was difficult; AI would help then.” |

*Note. Excerpts are translated from the original Korean responses and edited for clarity.*

**Table S2.** Effect sizes for the human–model comparisons by question type. Positive Cliff’s δ indicates the human rater scored higher than GPT-5 Thinking; 95% confidence intervals are from 10,000 bootstrap resamples. Kruskal–Wallis omnibus effects: Type A ε² = 0.075, Type B 0.052, Type C 0.091, Type D 0.054.

| **Type** | **Contrast (vs GPT-5 Thinking)** | **P (Dunn–Holm)** | **Cliff’s δ [95% CI]** | **Magnitude** |
| --- | --- | --- | --- | --- |
| A | Student 1 | >.99 | −0.02 [−0.18, +0.16] | negligible |
| A | Student 2 | .372 | −0.17 [−0.34, −0.01] | small |
| A | Resident 1 | .012 | +0.29 [+0.13, +0.44] | small |
| A | Resident 2 | .029 | +0.25 [+0.09, +0.41] | small |
| B | Student 1 | >.99 | −0.08 [−0.25, +0.09] | negligible |
| B | Student 2 | >.99 | −0.03 [−0.20, +0.14] | negligible |
| B | Resident 1 | .070 | +0.23 [+0.06, +0.39] | small |
| B | Resident 2 | .015 | +0.28 [+0.11, +0.44] | small |
| C | Student 1 | .387 | −0.15 [−0.31, +0.03] | negligible |
| C | Student 2 | .130 | −0.22 [−0.38, −0.05] | small |
| C | Resident 1 | .044 | +0.25 [+0.08, +0.41] | small |
| C | Resident 2 | .044 | +0.26 [+0.09, +0.42] | small |
| D | Student 1 | .164 | −0.15 [−0.28, −0.02] | small |
| D | Student 2 | .002 | −0.27 [−0.41, −0.13] | small |
| D | Resident 1 | <.001 | −0.35 [−0.49, −0.21] | medium |
| D | Resident 2 | .229 | −0.16 [−0.30, −0.02] | small |

**Table S3.** Reliability of the gold standard: agreement among the clinical reference raters (senior faculty member and two residents) on the clinical value of each answer.

| **Scope** | **% agreement** | **Krippendorff α** | **Gwet AC1** |
| --- | --- | --- | --- |
| Overall | 98.9 | 0.99 | 0.99 |
| Type A (general retrieval) | 100 | 1.00 | 1.00 |
| Type B (tooth/procedure) | 97.8 | 0.98 | 0.98 |
| Type C (interpretation) | 97.8 | 0.97 | 0.98 |
| Type D (absent information) | 100 | –* | 1.00 |

**On Type D items agreement is at the ceiling (e.g., all clinicians abstained), so the chance-corrected κ/α become unstable (the “kappa paradox”); Gwet's AC1 and percent agreement are used instead.*

**Table S4.** Agreement among all five human raters, including the two students (shown for completeness). Lower values reflect the students' developing competence rather than any weakness in the gold standard.

| **Scope** | **% agreement** | **Krippendorff α** | **Gwet AC1** |
| --- | --- | --- | --- |
| Overall | 85.8 | 0.86 | 0.83 |
| Type A (general retrieval) | 90.4 | 0.92 | 0.89 |
| Type B (tooth/procedure) | 83.7 | 0.85 | 0.80 |
| Type C (interpretation) | 78.9 | 0.79 | 0.71 |
| Type D (absent information) | 90.4 | 0.10* | 0.89 |

**On Type D items agreement is at the ceiling (e.g., all clinicians abstained), so the chance-corrected κ/α become unstable (the “kappa paradox”); Gwet's AC1 and percent agreement are used instead.*

**Table S5.** The model ranking is essentially unchanged when models are scored for clinical correctness rather than by SBERT (Spearman ρ=0.9, *P*=.001).

| **Model** | **Clinical accuracy (%)** | **SBERT median** |
| --- | --- | --- |
| GPT-5 Pro | 96.4 | 0.861 |
| GPT-5 Thinking | 95.6 | 0.900 |
| OpenAI o3 | 91.4 | 0.831 |
| OpenAI o4-mini | 84.4 | 0.695 |
| GPT-4.5 | 81.4 | 0.654 |
| GPT-4.1 | 80.6 | 0.623 |
| GPT-4o | 78.9 | 0.694 |
| GPT-5 (base) | 76.9 | 0.647 |
| GPT-4.1 mini | 68.9 | 0.529 |

**Table S6.** Performance on absent-information (Type D) items, where the correct response is to abstain. “Fabrication” denotes supplying a value the chart does not contain.

| **Rater / Model** | **Appropriate abstention (%)** | **Fabrication (%)** |
| --- | --- | --- |
| Clinicians (gold + 2 residents) | 100 | 0 |
| GPT-5 Pro | 97.8 | 2.2 |
| GPT-5 Thinking | 95.6 | 4.4 |
| OpenAI o3 | 86.7 | 13.3 |
| GPT-4o | 81.1 | 18.9 |
| GPT-4.1 | 81.1 | 18.9 |
| OpenAI o4-mini | 81.1 | 18.9 |
| GPT-4.5 | 73.3 | 26.7 |
| GPT-5 (base) | 71.1 | 28.9 |
| GPT-4.1 mini | 53.3 | 46.7 |

**Table S7.** Expert adjudication of clinically consequential discrepancies in responses generated by GPT-5 Thinking and GPT-5 Pro (n=59 adjudicated responses).

| **Subset stratum** | **Model** | **n** | **No clinical consequence (0), n** | **Minor discrepancy (1), n** | **Clinically consequential (2), n** |
| --- | --- | --- | --- | --- | --- |
| Content-incorrect (Types A–C)^a^ | GPT-5 Thinking | 12 | 4 | 7 | 1 |
|  | GPT-5 Pro | 11 | 3 | 5 | 3 |
| Type D fabrication | GPT-5 Thinking | 4 | 0 | 0 | 4 |
|  | GPT-5 Pro | 2 | 0 | 0 | 2 |
| Low-SBERT sample previously classified as content-correct^c^ | GPT-5 Thinking | 15 | 12 | 3 | 0 |
|  | GPT-5 Pro | 15 | 13 | 2 | 0 |
| **Total** |  | **59** | **32** | **17** | **10^b^** |

Two faculty dentists at a university dental hospital independently rated each response, blinded to model identity, automated scores, and subset stratum, using a three-level scale: 0, no clinical consequence (clinically equivalent to the gold standard or differing only in wording, format, or language); 1, minor discrepancy (an inaccuracy or omission unlikely to alter clinical interpretation or management in a supervised educational setting); 2, clinically consequential (could plausibly change chart interpretation or a management decision, or teaches an incorrect clinical fact, including fabricated information presented as fact). Exact agreement was 91.5% (54/59; Gwet AC1=0.879; linearly weighted κ=0.89), and all five disagreements were between adjacent categories and were resolved by consensus discussion.

^a^All Type A–C responses scored incorrect in the content-based clinical-accuracy re-scoring, regardless of SBERT similarity.

^b^The 10 identified cases represent 1.4% of the 720 outputs generated by the two models; because adjudication was subset-based, this percentage should not be interpreted as a full-sample incidence estimate.

^c^Random sample of 15 responses per model previously classified as content-correct with SBERT similarity <0.60, included to verify that low semantic similarity did not conceal clinically consequential errors; none was rated clinically consequential.
